# Anesthetic Challenges in Laparoscopic Surgery for a Child with Juvenile Dermatomyositis

**DOI:** 10.7759/cureus.71056

**Published:** 2024-10-08

**Authors:** Aruna Parameswari, S Kanishka, Keerthana Kalaimani, S K Saif Ali, Ganeshamoorthy Baskar

**Affiliations:** 1 Anesthesiology, Sri Ramachandra Institute of Higher Education and Research, Chennai, IND

**Keywords:** case report, juvenile dermatomyositis, laparoscopic surgery, supraglottic airway, transverse abdominis plane block

## Abstract

Conventional anesthetic management of juvenile dermatomyositis involves general anesthesia with endotracheal intubation using non-depolarizing muscle relaxants. Neuromuscular monitoring would be required for the titration of neuromuscular blocking drugs as these patients are more sensitive to these drugs, which leads to delayed recovery. Here we present a 14-year-old girl with juvenile dermatomyositis posted for laparoscopic appendicectomy. Considering the complications of the disease, ProSeal^TM^ laryngeal mask airway was used for airway management with ultrasound-guided transverse abdominis plane block for analgesia. This approach eliminated the need for non-depolarizing neuromuscular blockade and neuromuscular monitoring and provided adequate analgesia.

## Introduction

Juvenile dermatomyositis (JDM) is a systemic immune-mediated inflammatory myopathy involving the skin and muscles that affects children [1]. This is caused by CD4+, interferon 1 and T cell-mediated micro-vasculopathy, leading to perifascicular atrophy and muscle microinfarcts, which leads to decreased blood flow [2]. Proximal muscle weakness is a prominent symptom in children with juvenile dermatomyositis. Chronic calcium accumulation can lead to restriction of movement and joint contractures and they can also have residual skin lesions and muscle atrophy [3]. These patients have an abnormal response to depolarizing and non-depolarizing muscle relaxants [4-8]. Johns et al. reported an abnormal short-lived contracture in response to succinylcholine in a child with dermatomyositis [4]. You et al. noted a delay in recovery of limb muscle power with cis-atracurium in a patient with dermatomyositis [5]. Suzuki et al. observed a slow onset of action of rocuronium-induced neuromuscular block and a slow recovery of neuromuscular function with sugammadex in a patient with dermatomyositis [6]. Taking into account these findings and the lack of adequate data about patients with juvenile dermatomyositis, there are unique concerns in these patients such as delayed recovery from motor blockade with non-depolarizing muscle relaxants, poor cardiorespiratory effort, risk of hyperkalemia with succinylcholine, risk of pulmonary aspiration and need for postoperative mechanical ventilation [4-9]. In this case report, we describe the anesthetic management of a child with juvenile dermatomyositis undergoing laparoscopic appendicectomy. We used a second-generation supraglottic airway device for airway management and avoided the use of non-depolarizing neuromuscular blockade and worsening of neuromuscular weakness and managed postoperative pain with bilateral transverse abdominis plane block.

## Case presentation

A 14-year-old girl weighing 38 kg presented with complaints of dull aching abdominal pain and fever for two days. Computed tomography (CT) of the abdomen revealed acute appendicitis.

**Figure 1:**
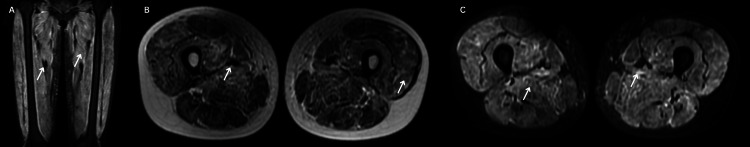
MRI imaging studies of thigh muscles. (A) Coronal T2-weighted STIR. (B) Axial T2 frFSE imaging. (C) Axial T2-weighted STIR showing signs of bilateral active severe myositis of both thighs with involvement of subcutaneous layer and fascia. STIR: Short tau inversion recovery; frFSE, fast relaxation fast spin echo sequence

She had a history of generalized erythematous scaly lesions over upper limbs, trunk and lower limbs, difficulty in standing up after sitting or squatting and difficulty in swallowing solid food for two months prior to the onset of abdominal pain. She was evaluated for neuromuscular disorders and found to have elevated creatinine phosphokinase, lactate dehydrogenase and C-reactive protein levels. Muscle biopsy and magnetic resonance imaging (MRI) of the thigh (Figure 1) revealed severe myositis and she was diagnosed with juvenile dermatomyositis. 

Preoperative airway examination demonstrated a mouth opening of more than 2 cm, mento-hyoid distance >4 cm and modified Mallampati Class I, and there was no restriction of neck movements. The motor power in all limbs was 4/5. Lab investigations (complete blood count, renal function test, serum electrolytes, coagulation profile) were normal. She had a breath-holding time of 25 seconds. During the preoperative evaluation, her chest X-ray and 12-lead electrocardiogram were normal. She was not started on any treatment for the dermatomyositis.

She was categorized under the American Society of Anesthesiologists (ASA) physical status class III for elective laparoscopic appendicectomy. She was fasted for solids and liquids for 8 hours preoperatively and was planned for general anesthesia with bilateral transverse abdominis plane (TAP) block. Informed consent was taken for surgery and anesthesia from the patient's guardian.

Pantaprazole 20mg was given intravenously 30 minutes before shifting the patient to the operation theater. Gastric ultrasound imaging was done in the preoperative holding area with the patient in the supine and right lateral position to visualize the contents of the gastric antrum and to assess the gastric antral volume, which was found to be less than 1.5 ml/kg.

Standard monitors, which included five-lead ECG, oxygen saturation (SpO_2_), non-invasive blood pressure and temperature, were attached. Anesthesia was induced with intravenous fentanyl 2 mcg/kg and propofol 2 mg/kg and a size 3 ProSeal^TM^ laryngeal mask airway (LMA) was placed and cuff inflated with 20 ml of air. The oropharyngeal leak pressure was 30 cm of H_2_O. A 10 French Ryle’s tube was inserted via the gastric port of the Proseal^TM^ LMA to allow continuous gastric drainage during the intraoperative period. Ultrasound-guided bilateral lateral transverse abdominis plane (TAP) block was given with 15 ml of 0.125% bupivacaine on each side using a high-frequency linear transducer. Paracetamol 15 mg/kg was given after securing the airway. Intraoperative anesthesia was maintained with sevoflurane (minimum alveolar concentration (MAC) of 0.8-1), air and oxygen (fraction of inspired oxygen (FiO_2_) of 0.35) with an end tidal CO_2_ of 35-40 mmHg on volume control ventilation. After insufflation of CO_2_ for laparoscopy, the airway pressure was between 25 and 28 cm of H_2_O with adequate tidal volume and a good capnography trace. The duration of the procedure was 40 minutes. At the end of the procedure, the supraglottic device was removed after the patient was fully awake.

Informed consent and written consent for publication was obtained from the patient’s guardian for this case report. This manuscript adheres to the CARE (CAse REports) guidelines (developed by an international group of experts) for case reports.

## Discussion

Conventional anesthetic management for laparoscopic appendicectomy involves general anesthesia with endotracheal intubation and muscle relaxants to provide adequate surgical conditions with neuromuscular monitoring for titration of muscle relaxants. Adequate neuromuscular blockade is especially essential in laparoscopic surgery as it reduces the insufflation pressure required while at the same time providing good surgical access [10]. 

**Figure 2:**
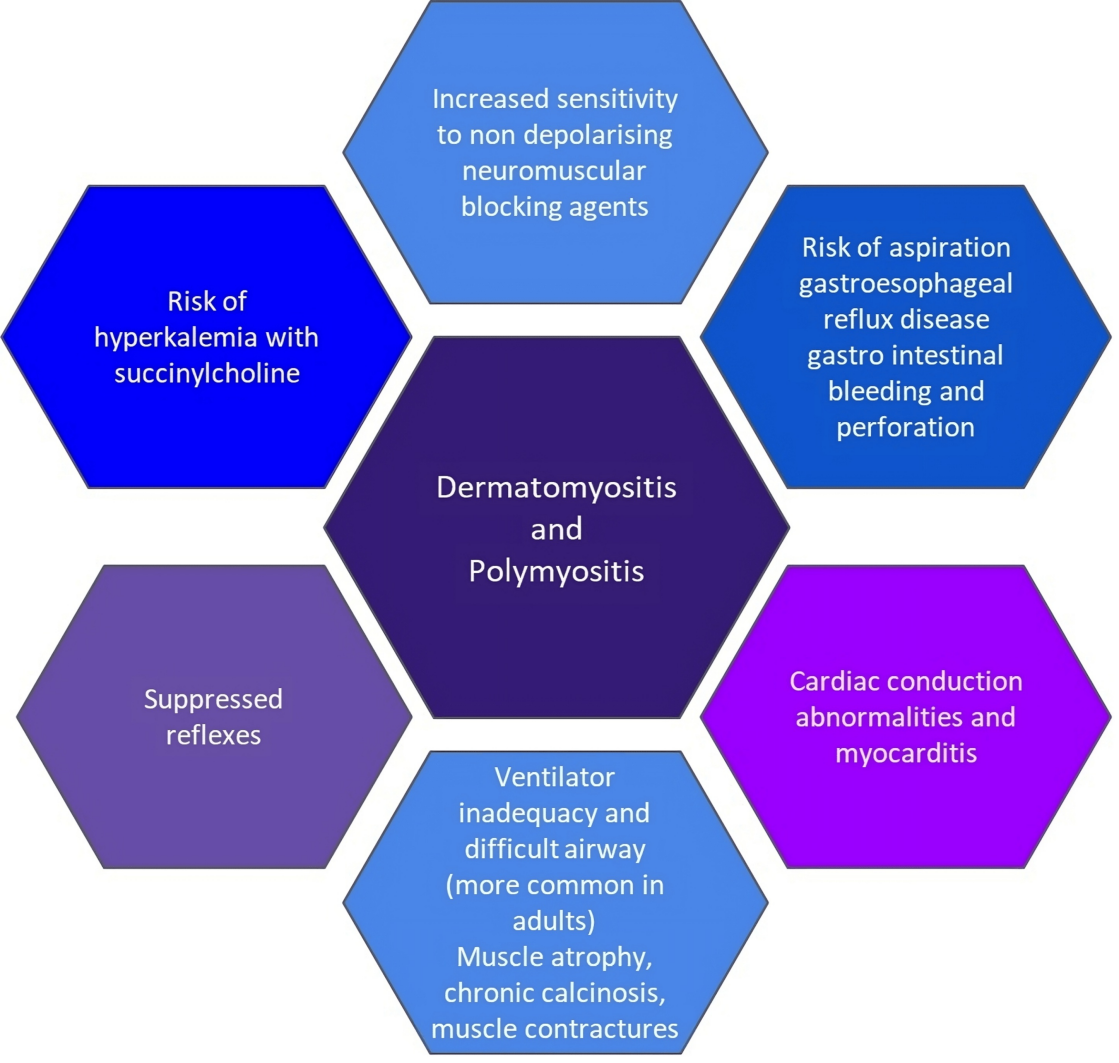
Anesthetic concerns in a child with juvenile dermatomyositis Image credits: Dr. Kanishka, Dr. Aruna Parameswari

The perioperative anesthesia concerns of a child with juvenile dermatomyositis are given in Figure 2.

Anesthetic concerns also include preoperative muscle weakness and excessive sensitivity to non-depolarizing muscle relaxants. This fact, along with the presence of poor cardiorespiratory reserve, puts them at increased risk of persistent muscle weakness after surgery, which can necessitate postoperative ventilatory support following normal intravenous muscle relaxant dosing. Our patient had proximal muscle weakness as evident from her inability to rise from a sitting position. Due to the pre-existent muscle weakness, good surgical conditions were obtained just with the use of sevoflurane and TAP block, even without the use of muscle relaxants.

Various studies support the use of a second-generation supraglottic airway as an alternative to the traditional endotracheal intubation in short-duration laparoscopy procedures [11,12]. ProSeal^TM^ is considered the gold standard second-generation supraglottic airway device that provides a good airway seal and has a gastric drainage tube.

Sinha et al. [12] demonstrated that the ProSeal^TM^ laryngeal mask airway provided adequate oropharyngeal seal and ventilation during elective laparoscopic procedures in children. Sinha et al. [13] and Halaseh et al. [14] conducted studies that proved the safe use of ProSeal^TM^ LMA even in patients with a higher risk of aspiration like obesity, pregnancy and laparoscopic procedures.

Our patient was adequately fasted preoperatively and in addition, we used a preoperative gastric ultrasound and measured the gastric antral cross-sectional area and we calculated the residual gastric volume, which was <1.5 ml/kg. It has been established in several studies that a gastric residual volume for liquids of <1.5 ml/kg can be considered safe with minimal risk of aspiration [15]. A Ryles tube was placed via the gastric port of the Proseal^TM^ LMA and intermittent suctioning was done intraoperatively.

Abdominal plane blocks like TAP blocks provide excellent intraoperative and postoperative analgesia, thus reducing opioid use. Here we used a ProSeal^TM^ LMA for airway management with bilateral TAP block for analgesia. Non-depolarizing muscle relaxant was avoided and an adequate plane of anesthesia was maintained with sevoflurane. There was minimal hemodynamic variation during the insufflation of carbon dioxide and in the postoperative period, the child did not have any complaint of pain for 12 hours. Surgeons did not have any difficulty during the procedure and the airway pressure was less than 28 mmHg. The patient was followed up for 24 hours. Bladder and bowel movements were normal and she was discharged uneventfully.

Other anesthetic options for this child would be total intravenous anesthesia and neuraxial anesthesia. There is no specific contraindication for neuraxial anesthesia in juvenile dermatomyositis and there are published case reports of surgery under neuraxial anesthesia in adults with dermatomyositis. However, considering this was a laparoscopic surgery on a 14-year-old, we did not consider neuraxial anesthesia as an option. Total intravenous anesthesia using target-controlled infusions of propofol and remifentanil can also be used safely.

## Conclusions

We conclude that a second-generation laryngeal mask airway and transverse abdominis plane block can be considered in the anesthetic management of a child with dermatomyositis for laparoscopic appendicectomy. This approach reduces the need for neuromuscular block and provides adequate postoperative analgesia. The supraglottic device has to be positioned correctly to ensure adequate ventilation and prevention of aspiration. The insertion of the gastric tube through the gastric channel with intermittent suction of this tube further ensures gastric decompression. With the expanding indications for the use of supraglottic devices, this case report is another evidence in support of the use of supraglottic devices for laparoscopic surgery in children with dermatomyositis.
